# Sex Differences in the Excess Risk of Cardiovascular Diseases Associated with Type 2 Diabetes: Potential Explanations and Clinical Implications

**DOI:** 10.1007/s12170-015-0462-5

**Published:** 2015-05-26

**Authors:** Sanne A. E. Peters, Rachel R. Huxley, Naveed Sattar, Mark Woodward

**Affiliations:** The George Institute for Global Health, Nuffield Department of Population Health, University of Oxford, 34 Broad Street, Oxford, OX1 3BD UK; School of Public Health, University of Queensland, Brisbane, Australia; Institute of Cardiovascular and Medical Sciences, University of Glasgow, Glasgow, UK; The George Institute for Global Health, University of Sydney, Sydney, Australia; Department of Epidemiology, Johns Hopkins University, Baltimore, MD USA

**Keywords:** Diabetes, Cardiovascular disease, Coronary heart disease, Stroke, Sex differences, Men, Women

## Abstract

Strong evidence suggests that type 2 diabetes confers a stronger excess risk of cardiovascular diseases in women than in men; with women having a 27 % higher relative risk of stroke and a 44 % higher relative risk of coronary heart disease compared with men. The mechanisms that underpin these sex differences in the associations between diabetes and cardiovascular disease risk are not fully understood. Some of the excess risk may be the result of a sex disparity in the management and treatment of diabetes, to the detriment of women. However, accruing evidence suggests that real biological differences between men and women underpin the excess risk of diabetes-related cardiovascular risk in women such that there is a greater decline in risk factor status in women than in men in the transition from normoglycemia to overt diabetes. This greater risk factor decline appears to be associated with women having to put on more weight than men, and thus attain a higher body mass index, to develop diabetes. Further studies addressing the mechanisms responsible for sex differences in the excess risk of cardiovascular diseases associated with diabetes are needed to improve the prevention and management of diabetes in clinical practise.

## Introduction

**Fig. 1:**
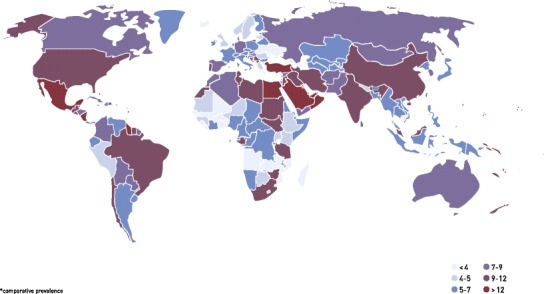
Prevalence (%) of diabetes in adults (20–79 years) in 2013. Figure is obtained from reference [1]

**Fig. 2:**
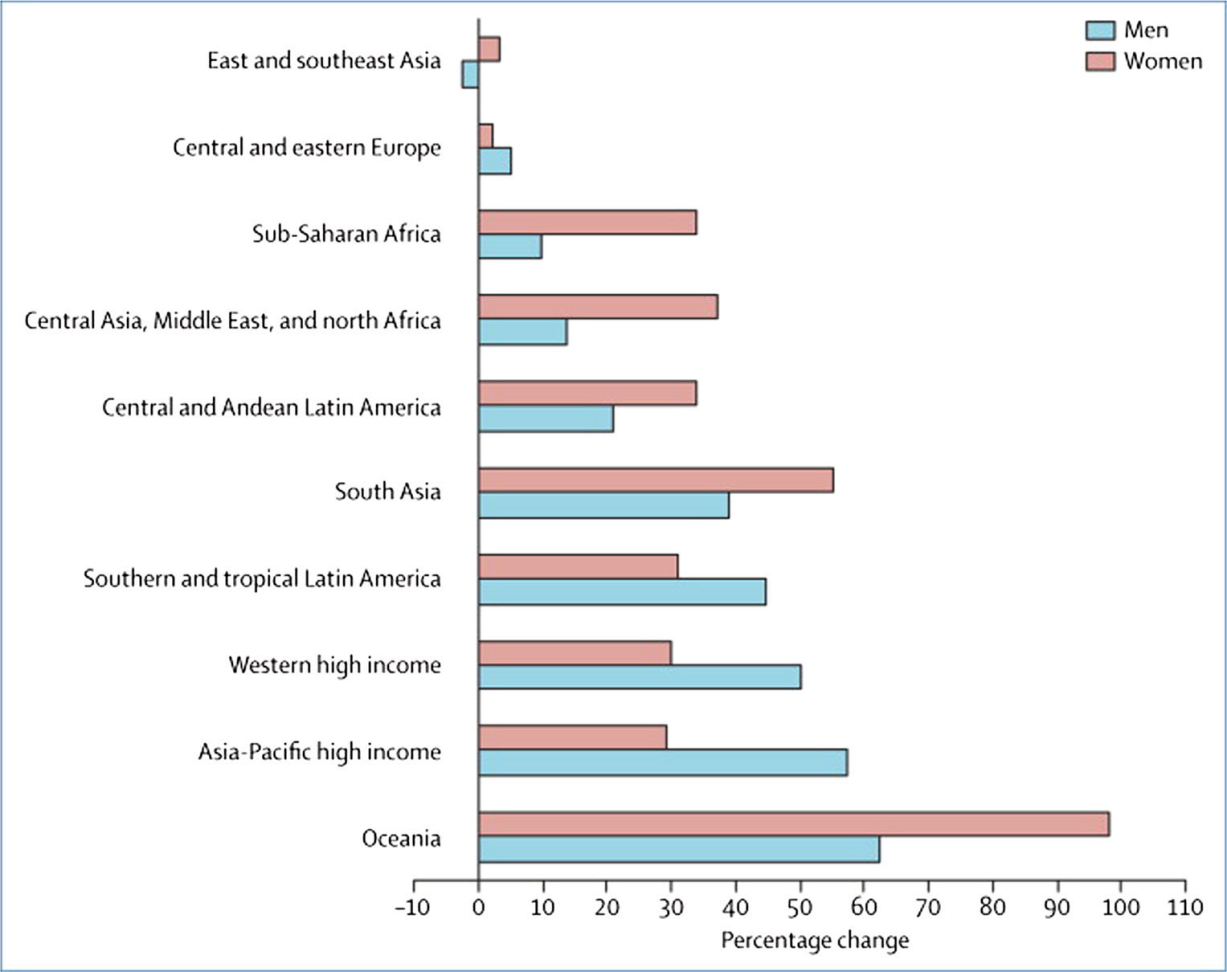
Percentage growth in age-standardised diabetes prevalence, 1980–2008, by region. Reproduced, with the permission of the publisher, from reference [3]

Diabetes mellitus is a major health concern; 382 million individuals or 8.3 % of the adult population worldwide have diabetes, and an additional 175 million people are unaware that they may have diabetes. Most people with diabetes live in low- and middle-income countries; countries in Oceania, North Africa and the Middle East have the highest prevalence of diabetes, with rates in these countries ranging from 21–25 % in men and 21–32 % in women (Fig. 1) [1, 2]. The prevalence of diabetes is expected to rise by 55 % to 592 million individuals by 2035 [1]; whilst much of this increase is driven by population growth and ageing, other key contributors are the rapid growth in the prevalence of overweight and obesity and the increasing lack of physical activity. Trends in the prevalence of diabetes vary substantially between regions and sexes, which is inherent to substantial biological, socioeconomic and societal differences (Fig. 2) [2, 3]. Type 2 diabetes is the most common type of diabetes and accounts for 85 to 95 % of all diabetes, with the remainder comprising mainly of type 1 diabetes—one of the most common autoimmune diseases.

Diabetes and its complications are major causes of early death in most countries. Approximately 5.1 million individuals died from diabetes in 2013, accounting for 8.4 % of global all-cause mortality amongst adults [1]. Although the global number of deaths due to diabetes is similar between men and women, there are important differences in the global distribution of these deaths [1]. About 25 % more men than women die of diabetes in the Western Pacific region, whereas diabetes accounts for 30 % more deaths in women than in men in Southeast Asia, and for over 50 % more deaths in women in Africa. This disparity may in part be due to higher rates of mortality from other causes, biological factors, or because of poorer access to healthcare amongst women in some regions.

Diabetes also poses a large economic and social burden on individuals and families, national health systems and countries. Health spending on diabetes accounted for 11 % of total health expenditure worldwide in 2013, equating to 548 billion USD and, unsurprisingly, only 20 % of this global health expenditure occurred in low- and middle-income countries, where about 80 % of all individuals with diabetes live [1]. Hence, whilst the impact of the diabetes epidemic is global, it disproportionally affects those living in socially and economically disadvantaged conditions.

### Sex Differences in the Excess Risk of Cardiovascular Diseases Associated with Type 2 Diabetes

**Fig. 3:**
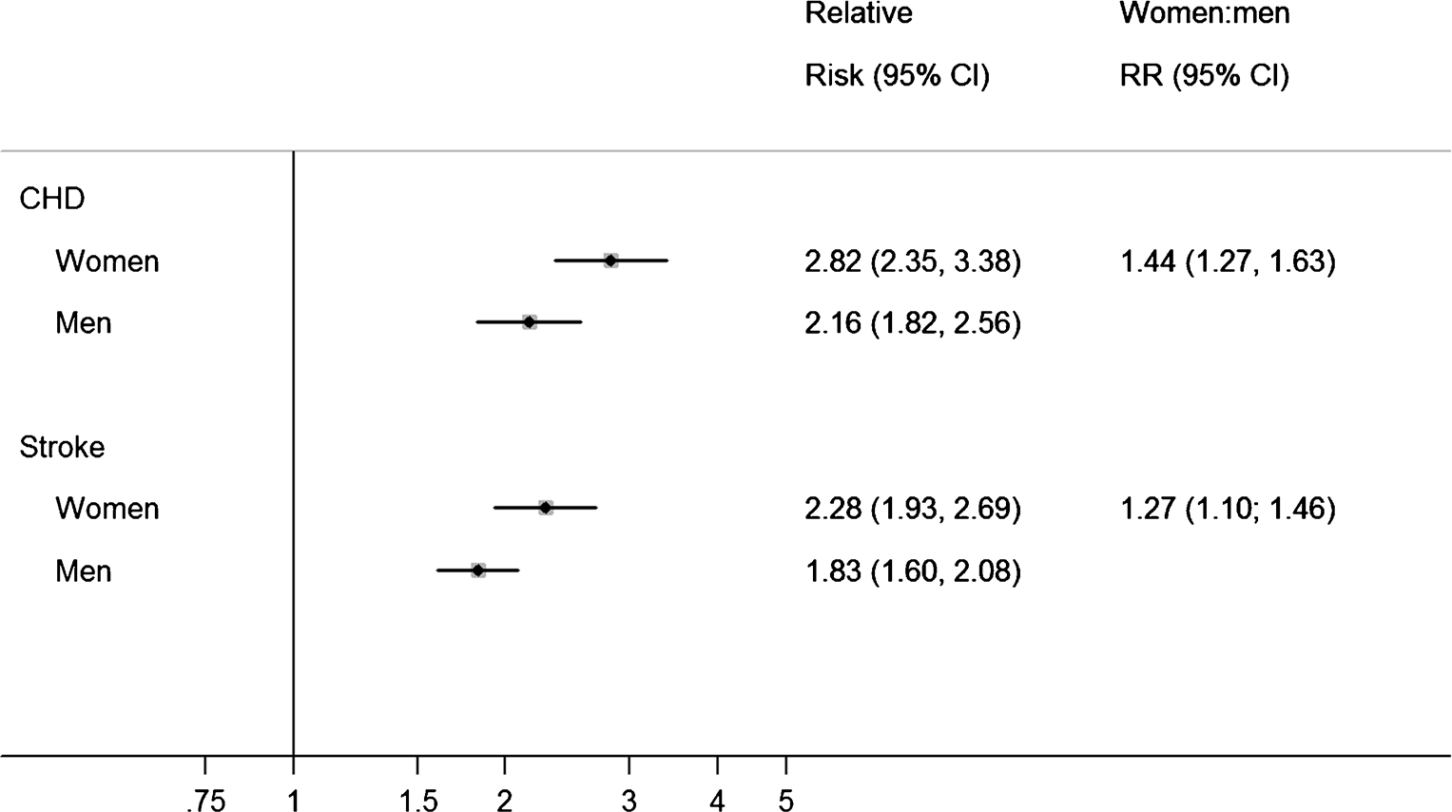
Relative risk (RR) and women:men ratio of relative risks (RRR) for coronary heart disease (CHD) and stroke in women and men with diabetes versus without diabetes. *Vertical bars* represent 95 % confidence intervals. Data were obtained from references [7•, 9•]

Considerable sex differences exist in the occurrence of the various manifestations of cardiovascular disease (CVD). Men have a higher risk of coronary heart disease (CHD), whereas women, who tend to have a longer life expectancy, have a similar or greater propensity of developing stroke [4, 5]. Cardiovascular disease is the most common underlying cause of death, accounting for 52 % of deaths in type 2 diabetes [6]. This estimate, however, is based on the assumption that the associations between diabetes and CVD outcomes are equivalent between women and men. An accruing body of literature shows that there are appreciable and clinically relevant differences in how diabetes affects the risk of CVD in men and women (Fig. 3). A recent pooled analysis summarising data from 64 cohorts, including nearly 900,000 individuals and over 28,000 incident CHD events, showed that the presence of diabetes nearly tripled the risk of incident CHD in women (RR 2.82 [95 % CI 2.35; 3.38]), whereas it little more than doubled the risk in men (RR 2.16 [95 % CI 1.82; 2.56]) [7•]. Therefore, diabetes conferred a 44 % (95 % CI 27–63 %) greater excess risk for incident CHD in women compared with men. This estimate is comparable to the 46 % excess risk for fatal CHD in women with diabetes found in a previous analysis [8]. In addition, a pooled analysis on data from 750,000 individuals and more than 12,000 incident stroke events provided strong evidence that women with diabetes mellitus have a 27 % (95 % CI 10, 46 %) greater increased risk of stroke compared with their male counterparts; the pooled relative risk of stroke associated with diabetes was 2.28 (95 % CI 1.93, 2.69) in women and 1.83 (95 % CI 1.60, 2.08) in men, independent of sex differences in other major cardiovascular risk factors [9•]. This sex differential was seen consistently across major predefined stroke, study and participant subtypes, which included a comparison of individuals from Asian and non-Asian populations. Furthermore, recent analyses of routinely collected health care data from the UK suggests that diabetes has a stronger association with the risk of CVD in women than in men; however, only amongst young people [10]. This may be explained by findings from previous large-scale studies that show that the effects of diabetes on the risk of CVD generally attenuate with age. Sex differences may continue to exist after the diagnosis of diabetes; a recent study suggested that the risk of stroke associated with higher HbA1c was greater in women than in men with type 2 diabetes; each 1 % increase in baseline HbA1c was associated with a 5 % (95 % CI 1.02, 1.07) higher relative risk of stroke in women and a 1 % (95 % CI 0.99, 1.04) higher relative risk of stroke in men [11].

### Sex Differences in the Management of Diabetes

A sex disparity in the management and treatment of cardiovascular risk factors in individuals with diabetes, to the detriment of women, possibly explains the excess risk of CVD in women with diabetes compared to men. Historically, women with diabetes were more likely to have a more adverse cardiovascular risk profile, were treated less aggressively and were less likely to achieve recommended levels of risk factors compared to male counterparts [12–16]. Whilst access to treatment has become more equitable between the sexes over the past decade, especially in high income countries, sex differences in medication use and risk factor control continue to exist [17, 18]. For example, the 2012–2013 report of the National Diabetes Audit on nearly 2 million individuals with diabetes in the UK showed that 58 % of women and 62 % of men with diabetes received recommended care processes, and that 34 % of women and 37 % of men achieved treatment targets for HbA1c, blood pressure and cholesterol [17]. After controlling for key confounding factors, such as age, ethnicity, deprivation group, body mass index (BMI) and duration of diabetes, women were 15 % less likely than men to meet all recommended care processes. Furthermore, other recent studies have shown that, even when treated similarly, women with diabetes are less likely than men to achieve target values for cardiovascular risk factors [19, 20]. We therefore hypothesise that sex differences in the management of diabetes alone are unlikely to explain all of the excess relative risk of CHD and stroke in women with diabetes. This is also supported by the results from the meta-analyses [7•, 8, 9•], which showed that the impact of accounting for levels of cardiovascular risk factors on the estimated excess risk of CHD and stroke associated with diabetes was similar in men and women.

### Sex Differences in Cardiovascular Risk Factors in the Development of Diabetes

Women without diabetes generally have more favourable levels of cardiovascular risk factors than men, but this pattern may reverse with a decline in glucose tolerance [8, 9•]. Several studies have shown that the deterioration in cardiovascular risk factor levels between individuals with and without type 2 diabetes is greater in women than in men (Table 1) [21–26]. The British Regional Heart Study and British Women’s Health and Heart Study found that women with diabetes had greater relative differences in many established and novel cardiovascular risk factors than men with diabetes including markers of coagulation, fibrinolysis, lipids and blood pressure, which were potentially mediated by greater differences in central adiposity and insulin resistance in women [23]. Hence, since many cardiovascular risk factors change to a greater extent in women than in men, women may have more cardiometabolic reserves and have to undergo greater metabolic deterioration to develop diabetes than do men. This hypothesis is supported by studies that found that levels of cardiovascular risk factors are already different between men and women before the conversion to a state of impaired glucose tolerance [27, 28]. Women, but not men, who progress from normoglycemia to pre-diabetes have higher levels of endothelial dysfunction, higher blood pressure and more abnormalities in their fibrinolysis and thrombosis pathways, compared to those who do not [28]. Therefore, the diabetes-related excess risk of CVD in women may not necessarily be due to any significant sex difference in the effects and complications of diabetes itself, but rather the result of a greater deterioration in cardiovascular risk factor levels in women compared to men in the transition to diabetes. Consequently, women diagnosed with diabetes may already be at a worse starting point than comparable men before treatment begins.

**Table 1:** Age-adjusted and multiple-adjusted mean difference^a^ (95 % confidence interval) in baseline risk factor levels amongst men and women with and without diabetes

Risk factor	Age-adjusted mean difference		Multiple-adjusted mean difference	
	Men	Women	Men	Women
Systolic blood pressure (mmHg)	5.33 (5.09; 5.58)	6.79 (6.40; 7.18)	4.18 (3.93; 4.44)	4.70 (4.26; 5.14)
Total cholesterol (mmol/L)	0.22 (0.20; 0.23)	0.24 (0.22; 0.27)	0.15 (0.13; 0.17)	0.14 (0.11; 0.17)
HDL cholesterol (mmol/L)	−0.09 (−0.11; −0.08)	−0.16 (−0.17; −0.14)	−0.06 (−0.08; −0.05)	−0.11 (−0.13; −0.09)
Body Mass Index (kg/m^2^)	1.01 (0.97; 1.06)	2.00 (1.91; 2.09)	0.64 (0.59; 0.68)	1.66 (1.57; 1.75)
Waist circumference (cm)	5.27 (4.79; 5.75)	9.06 (8.53; 9.59)	0.45 (0.21; 0.70)	2.20 (1.90; 2.50)

*HDL* high density lipoprotein cholesterol

^a^The difference is diabetes minus non-diabetes. Pooled data from the Asia Pacific Cohort Studies Collaboration, Atherosclerosis Risk in Communities Study, National Health and Nutrition Examination Survey III and Scottish Heart Health Extended Cohort. Multiple-adjusted mean differences are, where appropriate, adjusted for age, systolic blood pressure, smoking, body mass index and total cholesterol. Reproduced, with the permission of the publisher, from reference [9•]

### Sex Differences in Body Mass Index at the Time of Diagnosis

**Fig. 4:**
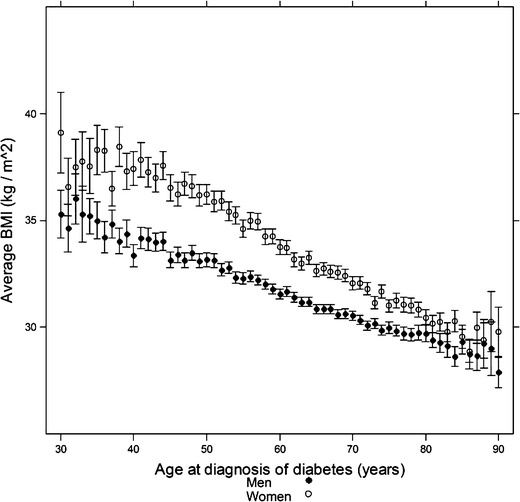
Mean body mass index by age at diagnosis of type 2 diabetes for men (*black*) and women (*white*) aged between 30 and 90 years at diagnosis and with BMI >25 kg/m^2^ from the Scottish Care Information Diabetes Collaboration (SCI-DC) dataset. Patients who died within 2 years of BMI determination were excluded. *Vertical bars* represent 95 % CIs around the mean. Reproduced, with the permission of the publisher, from reference [30]

Overweight and obesity is the key risk factor for the development of diabetes. Even though the magnitude of the association between BMI and diabetes is similar in men and women, studies have demonstrated that men need to attain a lower average BMI to be diagnosed with diabetes compared to women [29, 30]. For example, in a population-based diabetes register in Scotland, mean BMI closest to date of diagnosis of type 2 diabetes mellitus was 31.8 kg/m^2^ in men and 33.7 kg/m^2^ in women (Fig. 4) [30]. Results from the UK General Practice Research Database were almost identical to these Scottish findings; the age-adjusted average BMI at diagnosis of diabetes was nearly 2 kg/m^2^ higher in women than in men [29]. Both studies demonstrated that the difference in BMI at the time of diagnosis of diabetes was most marked at younger ages and narrowed with advancing age. Moreover, the Scottish data showed that HbA1c levels within 1 year of diagnoses were broadly similar in men and women, indicating that they were diagnosed at a similar stage of disease [30]. This implies that, at the same level of BMI, adult men without diabetes are more resistant to insulin than women or, similarly, that adult women are at lower risk of diabetes at an equivalent BMI.

The sex disparity in BMI at the time of diagnosis of diabetes can be linked to differential patterns of adiposity storage in men and women, and also to a healthier CVD risk profile in women without diabetes versus their male counterparts, as discussed above [31, 32•, 33]. Women, in general, have greater subcutaneous fat storage capacities and, linked to this, carry less visceral (i.e. more hazardous) fat than men. Since subcutaneous storage capacity is lower in men, excess adipose tissue is placed more rapidly into visceral and ectopic tissues, such as the liver and the skeletal muscle, which in turns leads to insulin resistance and interferes with insulin signalling pathways [34]. Women need to accumulate a greater amount of total adiposity, i.e. require attaining a higher level of BMI, than men for their subcutaneous storage to become exhausted, and to reach the same harmful visceral and ectopic fat deposition required to become insulin resistant and to develop diabetes [32•, 34]. Moreover, women, on average, tend to have a greater deterioration in metabolic risk factors including levels of blood pressure, lipids and inflammatory markers, and are likely to have accumulated more time living in a hazardous metabolic environment exacerbated by being in a pre-diabetic state compared with men in whom the deterioration in metabolic indices is less marked. This sex difference in the preferential location of fat storage (subcutaneous in women versus visceral/ectopic in men) and its associated metabolic changes may be crucial to the differential rates of development of diabetes (lower diabetes incidence in adult women) and, once diabetes ensues, to differential complication rates as discussed herein.

### Clinical Implications

Diabetes develops over decades; a recent study demonstrated that men, on average, have pre-diabetes for 8 years, and women for 10 years before they progress to overt diabetes [35]. This window presents an opportunity for identifying individuals at high risk for diabetes, and subsequently, for timely intervention to prevent or delay the onset of diabetes. The widening acceptance of diabetes diagnosis and its high risk states, via either fasting glucose (5.5 to 6.9 mmol/L as high risk) or HbA1c (6.0 to 6.4 % as high risk), has facilitated screening for, and diagnosis of, diabetes in clinical practise [36, 37]. HbA1c has the advantage of not requiring individuals to fast, enabling diagnosis to be done at any time and in any clinical situation, thus improving early detection of high risk for, or existing, diabetes. What is needed now is better facilitation of lifestyle changes to help men and women at risk to favourably change their weight, or weight trajectory, to delay or prevent the onset of diabetes. There is evidence that commercial weight loss companies do better than routine clinical services in helping people to lose weight and that women, in particular, are more comfortable with such services [38]. Similarly, increased awareness of the magnitude and timing of the risk of diabetes after gestational diabetes could provide an opportunity to facilitate lifestyle interventions that might prevent or delay the onset of type 2 diabetes in affected women [39, 40]. Regular monitoring of HbA1c in women with gestational diabetes will help facilitate risk screening. As cardiovascular risk factors escalate more in women as they transition to diabetes, physicians should take particular care to conduct a comprehensive cardiovascular risk assessment in women (whilst not neglecting men) noted to be at elevated risk of diabetes. These ideas fit with the need, wherever possible, to combine risk assessments for CVD and diabetes in primary care, in simple and pragmatic ways, as recently argued [41]. Finally, all physicians should be made aware that development of diabetes is associated with a greater increase in cardiovascular risk in women than in men so that they should, at the very least, treat women with diabetes as aggressively as they do with their male counterparts.

## Conclusion

There is accumulating evidence that the impact of type 2 diabetes on cardiovascular risk differs profoundly between the sexes and is more hazardous for women than for men. Although this may in part reflect a treatment disparity between women and men with diabetes, there are several lines of evidence to suggest that real behavioural and biological sex differences exist which may underpin the excess risk of diabetes-related cardiovascular risk in women. A greater deterioration in risk factor status in women than in men as they transition to diabetes, before the development of overt diabetes, appears to play a crucial role. This greater risk factor decline may in turn be related to women having to put on more weight than men (and thus attain a higher BMI) to develop diabetes. Future work aimed further clarification and understanding of the mechanisms responsible for sex differences in the excess risk of cardiovascular diseases associated with diabetes will be needed to improve the prevention and management of diabetes in clinical practise.

## References

[1] International Diabetes Federation (2013). IDF diabetes atlas.

[2] Danaei G, Finucane MM, Lu Y (2011). National, regional, and global trends in fasting plasma glucose and diabetes prevalence since 1980: systematic analysis of health examination surveys and epidemiological studies with 370 country-years and 2.7 million participants. Lancet.

[3] Tobias M (2011). Global control of diabetes: information for action. Lancet.

[4] Mozaffarian D, Benjamin EJ, Go AS (2015). Heart disease and stroke statistics—2015 update: a report from the american heart association. Circulation.

[5] Leening MJ, Ferket BS, Steyerberg EW (2014). Sex differences in lifetime risk and first manifestation of cardiovascular disease: prospective population based cohort study. BMJ (Clin Res ed).

[6] Morrish NJ, Wang SL, Stevens LK, Fuller JH, Keen H (2001). Mortality and causes of death in the WHO multinational study of vascular disease in diabetes. Diabetologia.

[7.•] Peters SA, Huxley RR, Woodward M (2014). Diabetes as risk factor for incident coronary heart disease in women compared with men: a systematic review and meta-analysis of 64 cohorts including 858,507 individuals and 28,203 coronary events. Diabetologia.

[8] Huxley R, Barzi F, Woodward M (2006). Excess risk of fatal coronary heart disease associated with diabetes in men and women: meta-analysis of 37 prospective cohort studies. BMJ (Clin Res ed).

[9.•] Peters SA, Huxley RR, Woodward M (2014). Diabetes as a risk factor for stroke in women compared with men: a systematic review and meta-analysis of 64 cohorts, including 775,385 individuals and 12,539 strokes. Lancet.

[10] Shah AD, Langenberg C, Rapsomaniki E (2014). Type 2 diabetes and incidence of cardiovascular diseases: a cohort study in 1.9 million people. Lancet Diabetes Endocrinol.

[11] Zhao W, Katzmarzyk PT, Horswell R, Wang Y, Johnson J, Hu G (2014). Sex differences in the risk of stroke and HbA(1c) among diabetic patients. Diabetologia.

[12] Ferrara A, Mangione CM, Kim C (2008). Sex disparities in control and treatment of modifiable cardiovascular disease risk factors among patients with diabetes: Translating Research Into Action for Diabetes (TRIAD) Study. Diabetes Care.

[13] Gouni-Berthold I, Berthold HK, Mantzoros CS, Bohm M, Krone W (2008). Sex disparities in the treatment and control of cardiovascular risk factors in type 2 diabetes. Diabetes Care.

[14] Penno G, Solini A, Bonora E (2013). Gender differences in cardiovascular disease risk factors, treatments and complications in patients with type 2 diabetes: the RIACE Italian multicentre study. J Intern Med.

[15] Wexler DJ, Grant RW, Meigs JB, Nathan DM, Cagliero E (2005). Sex disparities in treatment of cardiac risk factors in patients with type 2 diabetes. Diabetes Care.

[16] Kramer HU, Raum E, Ruter G (2012). Gender disparities in diabetes and coronary heart disease medication among patients with type 2 diabetes: results from the DIANA study. Cardiovasc Diabetol.

[17] Health and Social Care Information Centre. National diabetes audit 2012–2013 - report 1: care processes and treatment targets; 2014.

[18] Manteuffel M, Williams S, Chen W, Verbrugge RR, Pittman DG, Steinkellner A (2014). Influence of patient sex and gender on medication use, adherence, and prescribing alignment with guidelines. J Womens Health (2002).

[19] Franzini L, Ardigo D, Cavalot F (2013). Women show worse control of type 2 diabetes and cardiovascular disease risk factors than men: results from the MIND.IT Study Group of the Italian Society of Diabetology. Nutr Metab Cardiovasc Dis NMCD.

[20] Winston GJ, Barr RG, Carrasquillo O, Bertoni AG, Shea S (2009). Sex and racial/ethnic differences in cardiovascular disease risk factor treatment and control among individuals with diabetes in the Multi-Ethnic Study of Atherosclerosis (MESA). Diabetes Care.

[21] Collier A, Ghosh S, Hair M, Waugh N. Impact of socioeconomic status and gender on glycaemic control, cardiovascular risk factors and diabetes complications in type 1 and 2 diabetes: a population based analysis from a Scottish region. Diabetes Metab. 2014. 10.1016/j.diabet.2014.09.004 25454092

[22] Mascarenhas-Melo F, Marado D, Palavra F (2013). Diabetes abrogates sex differences and aggravates cardiometabolic risk in postmenopausal women. Cardiovasc Diabetol.

[23] Wannamethee SG, Papacosta O, Lawlor DA (2012). Do women exhibit greater differences in established and novel risk factors between diabetes and non-diabetes than men? The British Regional Heart Study and British Women’s Heart Health Study. Diabetologia.

[24] Steinberg HO, Paradisi G, Cronin J (2000). Type II diabetes abrogates sex differences in endothelial function in premenopausal women. Circulation.

[25] Mansfield MW, Heywood DM, Grant PJ (1996). Sex differences in coagulation and fibrinolysis in white subjects with non-insulin-dependent diabetes mellitus. Arterioscler Thromb Vasc Biol.

[26] Howard BV, Cowan LD, Go O, Welty TK, Robbins DC, Lee ET (1998). Adverse effects of diabetes on multiple cardiovascular disease risk factors in women. The Strong Heart Study. Diabetes Care.

[27] Haffner SM, Miettinen H, Stern MP (1997). Relatively more atherogenic coronary heart disease risk factors in prediabetic women than in prediabetic men. Diabetologia.

[28] Donahue RP, Rejman K, Rafalson LB, Dmochowski J, Stranges S, Trevisan M (2007). Sex differences in endothelial function markers before conversion to pre-diabetes: does the clock start ticking earlier among women? The Western New York Study. Diabetes Care.

[29] Paul S, Thomas G, Majeed A, Khunti K, Klein K (2012). Women develop type 2 diabetes at a higher body mass index than men. Diabetologia.

[30] Logue J, Walker JJ, Colhoun HM (2011). Do men develop type 2 diabetes at lower body mass indices than women?. Diabetologia.

[31] Geer EB, Shen W (2009). Gender differences in insulin resistance, body composition, and energy balance. Gend Med.

[32.•] Sattar N (2013). Gender aspects in type 2 diabetes mellitus and cardiometabolic risk. Best Pract Res Clin Endocrinol Metab.

[33] Garaulet M, Perex-Llamas F, Fuente T, Zamora S, Tebar FJ (2000). Anthropometric, computed tomography and fat cell data in an obese population: relationship with insulin, leptin, tumor necrosis factor-alpha, sex hormone-binding globulin and sex hormones. Eur J Endocrinol Eur Fed Endocr Soc.

[34] Sattar N, Gill JM (2014). Type 2 diabetes as a disease of ectopic fat?. BMC Med.

[35] Bertram MY, Vos T (2010). Quantifying the duration of pre-diabetes. Aust N Z J Public Health.

[36] Chatterton H, Younger T, Fischer A, Khunti K (2012). Risk identification and interventions to prevent type 2 diabetes in adults at high risk: summary of NICE guidance. BMJ (Clin Res ed).

[37] Ryden L, Grant PJ, Anker SD (2013). ESC Guidelines on diabetes, pre-diabetes, and cardiovascular diseases developed in collaboration with the EASD: the task force on diabetes, pre-diabetes, and cardiovascular diseases of the European Society of Cardiology (ESC) and developed in collaboration with the European Association for the Study of Diabetes (EASD). Eur Heart J.

[38] Jebb SA, Ahern AL, Olson AD (2011). Primary care referral to a commercial provider for weight loss treatment versus standard care: a randomised controlled trial. Lancet.

[39] Baptiste-Roberts K, Barone BB, Gary TL (2009). Risk factors for type 2 diabetes among women with gestational diabetes: a systematic review. Am J Med.

[40] Bellamy L, Casas JP, Hingorani AD, Williams D (2009). Type 2 diabetes mellitus after gestational diabetes: a systematic review and meta-analysis. Lancet.

[41] Preiss D, Khunti K, Sattar N (2011). Combined cardiovascular and diabetes risk assessment in primary care. Diabet Med J Br Diabet Assoc.

